# Supplementary material for: Hybrid Models and Biological Model Reduction with PyDSTool
Source: PLoS Comput Biol. 2012 Aug 9;8(8):e1002628. doi: 10.1371/journal.pcbi.1002628 (PMC3415397; doi:10.1371/journal.pcbi.1002628)
Supplement: Text S4 — Complete source code for the PyDSTool package (version 0.88.120504). Includes API documentation and help files linking to web pages. This file is identical to the current public release on Sourceforge.net. (ZIP) [file pcbi.1002628.s004.zip › PyDSTool/html/PyDSTool.Toolbox.adjointPRC-module.html]

xml version="1.0" encoding="ascii"?


PyDSTool.Toolbox.adjointPRC


| Home | Trees | Indices | Help | PyDSTool |
| --- | --- | --- | --- | --- |

Package PyDSTool ::
Package Toolbox ::
Module adjointPRC

[hide private]

[frames] | no frames]

# Module adjointPRC

source code


|  |  |
| --- | --- |
| Functions | [hide private] |

|  |  |
| --- | --- |
| adjointPRC(model, limit\_cycle, vname, freepar, numIntervals=500, numCollocation=7, method=`'``standard``'`, spike\_est=None, saveData=False, doPlot=False, verbosity=0, fname=None, force=False)  Create adjoint PRC from a limit cycle using AUTO, given an ODE\_Generator or Model object. | source code |

|  |  |
| --- | --- |
| rotate\_phase(pts, phase0\_ix)  Phase shift a pointset (assumed to be a cycle) about index phase0\_ix, i.e. | source code |

|  |  |
| --- | --- |
| find\_zero\_phase(prc, est\_ix)  prc should be of a single variable of interest | source code |


|  |  |
| --- | --- |
| Variables | [hide private] |

ALLOW\_THREADS = `1`

Abs = `Abs (ModelSpec wrapper)`

Acos = `Acos (ModelSpec wrapper)`

Asin = `Asin (ModelSpec wrapper)`

Atan = `Atan (ModelSpec wrapper)`

Atan2 = `Atan2 (ModelSpec wrapper)`

BUFSIZE = `10000`

Betavariate = `Betavariate (ModelSpec wrapper)`

CLIP = `0`

Ceil = `Ceil (ModelSpec wrapper)`

Choice = `Choice (ModelSpec wrapper)`

Cos = `Cos (ModelSpec wrapper)`

Cosh = `Cosh (ModelSpec wrapper)`

Degrees = `Degrees (ModelSpec wrapper)`

E = `QuantSpec e (ExpFuncSpec)`

ERR\_CALL = `3`

ERR\_DEFAULT = `0`

ERR\_DEFAULT2 = `2084`

ERR\_IGNORE = `0`

ERR\_LOG = `5`

ERR\_PRINT = `4`

ERR\_RAISE = `2`

ERR\_WARN = `1`

Exp = `Exp (ModelSpec wrapper)`

Expovariate = `Expovariate (ModelSpec wrapper)`

FLOATING\_POINT\_SUPPORT = `1`

FPE\_DIVIDEBYZERO = `1`

FPE\_INVALID = `8`

FPE\_OVERFLOW = `2`

FPE\_UNDERFLOW = `4`

Fabs = `Fabs (ModelSpec wrapper)`

False\_ = `False`

Floor = `Floor (ModelSpec wrapper)`

Fmod = `Fmod (ModelSpec wrapper)`

Frexp = `Frexp (ModelSpec wrapper)`

Gammavariate = `Gammavariate (ModelSpec wrapper)`

Gauss = `Gauss (ModelSpec wrapper)`

Getrandbits = `Getrandbits (ModelSpec wrapper)`

Getstate = `Getstate (ModelSpec wrapper)`

Hypot = `Hypot (ModelSpec wrapper)`

Infinity = `inf`

Jumpahead = `Jumpahead (ModelSpec wrapper)`

Ldexp = `Ldexp (ModelSpec wrapper)`

Log = `Log (ModelSpec wrapper)`

Log10 = `Log10 (ModelSpec wrapper)`

Lognormvariate = `Lognormvariate (ModelSpec wrapper)`

MAXDIMS = `32`

Max = `Max (ModelSpec wrapper)`

Min = `Min (ModelSpec wrapper)`

Modf = `Modf (ModelSpec wrapper)`

NAN = `nan`

NINF = `-inf`

NZERO = `-0.0`

Normalvariate = `Normalvariate (ModelSpec wrapper)`

PINF = `inf`

PZERO = `0.0`

Paretovariate = `Paretovariate (ModelSpec wrapper)`

Pi = `QuantSpec pi (ExpFuncSpec)`

Pow = `Pow (ModelSpec wrapper)`

RAISE = `2`

Radians = `Radians (ModelSpec wrapper)`

Randint = `Randint (ModelSpec wrapper)`

Random = `Random (ModelSpec wrapper)`

Randrange = `Randrange (ModelSpec wrapper)`

SHIFT\_DIVIDEBYZERO = `0`

SHIFT\_INVALID = `9`

SHIFT\_OVERFLOW = `3`

SHIFT\_UNDERFLOW = `6`

Sample = `Sample (ModelSpec wrapper)`

ScalarType = `(<type 'int'>, <type 'float'>, <type 'complex'>, ...`

Seed = `Seed (ModelSpec wrapper)`

Setstate = `Setstate (ModelSpec wrapper)`

Shuffle = `Shuffle (ModelSpec wrapper)`

Sin = `Sin (ModelSpec wrapper)`

Sinh = `Sinh (ModelSpec wrapper)`

Sqrt = `Sqrt (ModelSpec wrapper)`

Sum = `Sum (ModelSpec wrapper)`

Systemrandom = `Systemrandom (ModelSpec wrapper)`

Tan = `Tan (ModelSpec wrapper)`

Tanh = `Tanh (ModelSpec wrapper)`

True\_ = `True`

UFUNC\_BUFSIZE\_DEFAULT = `10000`

UFUNC\_PYVALS\_NAME = `'UFUNC_PYVALS'`

Uniform = `Uniform (ModelSpec wrapper)`

Vonmisesvariate = `Vonmisesvariate (ModelSpec wrapper)`

WRAP = `1`

Weibullvariate = `Weibullvariate (ModelSpec wrapper)`

Wichmannhill = `Wichmannhill (ModelSpec wrapper)`

absolute = `<ufunc 'absolute'>`

add = `<ufunc 'add'>`

bitwise\_and = `<ufunc 'bitwise_and'>`

bitwise\_not = `<ufunc 'invert'>`

bitwise\_or = `<ufunc 'bitwise_or'>`

bitwise\_xor = `<ufunc 'bitwise_xor'>`

c\_ = `<numpy.lib.index_tricks.CClass object at 0x1154330>`

cast = `{<type 'numpy.int64'>: <function <lambda> at 0x10a8bb0>...`

conj = `<ufunc 'conjugate'>`

conjugate = `<ufunc 'conjugate'>`

copysign = `<ufunc 'copysign'>`

deg2rad = `<ufunc 'deg2rad'>`

divide = `<ufunc 'divide'>`

equal = `<ufunc 'equal'>`

exp2 = `<ufunc 'exp2'>`

expm1 = `<ufunc 'expm1'>`

floor\_divide = `<ufunc 'floor_divide'>`

fmax = `<ufunc 'fmax'>`

fmin = `<ufunc 'fmin'>`

greater\_equal = `<ufunc 'greater_equal'>`

index\_exp = `<numpy.lib.index_tricks.IndexExpression object at ...`

inf = `inf`

infty = `inf`

invert = `<ufunc 'invert'>`

isinf = `<ufunc 'isinf'>`

left\_shift = `<ufunc 'left_shift'>`

little\_endian = `True`

log1p = `<ufunc 'log1p'>`

logaddexp = `<ufunc 'logaddexp'>`

logaddexp2 = `<ufunc 'logaddexp2'>`

logical\_and = `<ufunc 'logical_and'>`

logical\_not = `<ufunc 'logical_not'>`

logical\_xor = `<ufunc 'logical_xor'>`

maximum = `<ufunc 'maximum'>`

mgrid = `<numpy.lib.index_tricks.nd_grid object at 0x113d490>`

minimum = `<ufunc 'minimum'>`

multiply = `<ufunc 'multiply'>`

n = `9`

nan = `nan`

nbytes = `{<type 'numpy.int64'>: 8, <type 'numpy.int16'>: 2, <t...`

negative = `<ufunc 'negative'>`

newaxis = `None`

nextafter = `<ufunc 'nextafter'>`

not\_equal = `<ufunc 'not_equal'>`

ogrid = `<numpy.lib.index_tricks.nd_grid object at 0x113dbb0>`

ones\_like = `<ufunc 'ones_like'>`

r\_ = `<numpy.lib.index_tricks.RClass object at 0x11542f0>`

rad2deg = `<ufunc 'rad2deg'>`

reciprocal = `<ufunc 'reciprocal'>`

remainder = `<ufunc 'remainder'>`

right\_shift = `<ufunc 'right_shift'>`

rint = `<ufunc 'rint'>`

s\_ = `<numpy.lib.index_tricks.IndexExpression object at 0x11543f0>`

sctypeDict = `{0: <type 'numpy.bool_'>, 1: <type 'numpy.int8'>,...`

sctypeNA = `{'?': 'Bool', 'B': 'UInt8', 'Bool': <type 'numpy.bo...`

sctypes = `{'complex': [<type 'numpy.complex64'>, <type 'numpy....`

signbit = `<ufunc 'signbit'>`

spacing = `<ufunc 'spacing'>`

square = `<ufunc 'square'>`

subtract = `<ufunc 'subtract'>`

t = `'0'`

true\_divide = `<ufunc 'true_divide'>`

trunc = `<ufunc 'trunc'>`

typeDict = `{0: <type 'numpy.bool_'>, 1: <type 'numpy.int8'>, 2...`

typeNA = `{'?': 'Bool', 'B': 'UInt8', 'Bool': <type 'numpy.bool...`

typecodes = `{'All': '?bhilqpBHILQPfdgFDGSUVOMm', 'AllFloat': '...`


|  |  |
| --- | --- |
| Function Details | [hide private] |

|  |  |
| --- | --- |
| adjointPRC(model, limit\_cycle, vname, freepar, numIntervals=500, numCollocation=7, method=`'``standard``'`, spike\_est=None, saveData=False, doPlot=False, verbosity=0, fname=None, force=False) | source code |

```
Create adjoint PRC from a limit cycle using AUTO, given an
ODE_Generator or Model object.

spike_est (default None) provides an estimate of the spike time in
the limit cycle, if applicable. If set, this can be useful to
ensure the PRC is phase-shifted to start at the associated zero
(in the variable given by vname).

method: 'standard' is currently the only working method for calculating
  the eigenvetors corresponding to the unit eigenvalue. 'cyclic' selects
  the cyclic method.

Optionally save the PRC data and make plots.
Returns a dictionary containing the PRC, cycles with independent
variable values scaled to [0,1], the Jacobian, the flow maps,
the eigenvector corresponding to the unit eigenvalue, and
other algorithmic information.
```

|  |  |
| --- | --- |
| rotate\_phase(pts, phase0\_ix) | source code |

Phase shift a pointset (assumed to be a cycle) about index phase0\_ix,
i.e. 'rotate' it to put the point at phase0\_ix at the beginning of the
pointset.

NOTE: Does not update any label info that might be attached to
pts!

  


|  |  |
| --- | --- |
| Variables Details | [hide private] |

### ScalarType

Value:
:   ```
    (<type 'int'>,
     <type 'float'>,
     <type 'complex'>,
     <type 'long'>,
     <type 'bool'>,
     <type 'str'>,
     <type 'unicode'>,
     <type 'buffer'>,
    ...
    ```

### cast

Value:
:   ```
    {<type 'numpy.int64'>: <function <lambda> at 0x10a8bb0>, <type 'numpy.
    int16'>: <function <lambda> at 0x10a8bf0>, <type 'numpy.complex128'>: 
    <function <lambda> at 0x10a8c30>, <type 'numpy.uint64'>: <function <la
    mbda> at 0x10a8c70>, <type 'numpy.complex256'>: <function <lambda> at 
    0x10a8cf0>, <type 'numpy.float32'>: <function <lambda> at 0x10a8d30>, 
    <type 'numpy.bool_'>: <function <lambda> at 0x10a8cb0>, <type 'numpy.u
    int8'>: <function <lambda> at 0x10a8db0>, <type 'numpy.int32'>: <funct
    ion <lambda> at 0x10a8eb0>, <type 'numpy.int8'>: <function <lambda> at
    ...
    ```

### index\_exp

Value:
:   ```
    <numpy.lib.index_tricks.IndexExpression object at 0x11543b0>
    ```

### nbytes

Value:
:   ```
    {<type 'numpy.int64'>: 8, <type 'numpy.int16'>: 2, <type 'numpy.comple
    x128'>: 16, <type 'numpy.uint64'>: 8, <type 'numpy.bool_'>: 1, <type '
    numpy.complex256'>: 32, <type 'numpy.float32'>: 4, <type 'numpy.int8'>
    : 1, <type 'numpy.uint8'>: 1, <type 'numpy.uint16'>: 2, <type 'numpy.o
    bject_'>: 4, <type 'numpy.float64'>: 8, <type 'numpy.int32'>: 4, <type
     'numpy.string_'>: 0, <type 'numpy.void'>: 0, <type 'numpy.float128'>:
     16, <type 'numpy.int32'>: 4, <type 'numpy.uint32'>: 4, <type 'numpy.u
    nicode_'>: 0, <type 'numpy.complex64'>: 8, <type 'numpy.uint32'>: 4}
    ```

### sctypeDict

Value:
:   ```
    {0: <type 'numpy.bool_'>,
     1: <type 'numpy.int8'>,
     2: <type 'numpy.uint8'>,
     3: <type 'numpy.int16'>,
     4: <type 'numpy.uint16'>,
     5: <type 'numpy.int32'>,
     6: <type 'numpy.uint32'>,
     7: <type 'numpy.int32'>,
    ...
    ```

### sctypeNA

Value:
:   ```
    {'?': 'Bool',
     'B': 'UInt8',
     'Bool': <type 'numpy.bool_'>,
     'Complex128': <type 'numpy.complex256'>,
     'Complex32': <type 'numpy.complex64'>,
     'Complex64': <type 'numpy.complex128'>,
     'D': 'Complex64',
     'F': 'Complex32',
    ...
    ```

### sctypes

Value:
:   ```
    {'complex': [<type 'numpy.complex64'>,
                 <type 'numpy.complex128'>,
                 <type 'numpy.complex256'>],
     'float': [<type 'numpy.float32'>,
               <type 'numpy.float64'>,
               <type 'numpy.float128'>],
     'int': [<type 'numpy.int8'>,
             <type 'numpy.int16'>,
    ...
    ```

### typeDict

Value:
:   ```
    {0: <type 'numpy.bool_'>,
     1: <type 'numpy.int8'>,
     2: <type 'numpy.uint8'>,
     3: <type 'numpy.int16'>,
     4: <type 'numpy.uint16'>,
     5: <type 'numpy.int32'>,
     6: <type 'numpy.uint32'>,
     7: <type 'numpy.int32'>,
    ...
    ```

### typeNA

Value:
:   ```
    {'?': 'Bool',
     'B': 'UInt8',
     'Bool': <type 'numpy.bool_'>,
     'Complex128': <type 'numpy.complex256'>,
     'Complex32': <type 'numpy.complex64'>,
     'Complex64': <type 'numpy.complex128'>,
     'D': 'Complex64',
     'F': 'Complex32',
    ...
    ```

### typecodes

Value:
:   ```
    {'All': '?bhilqpBHILQPfdgFDGSUVOMm',
     'AllFloat': 'fdgFDG',
     'AllInteger': 'bBhHiIlLqQpP',
     'Character': 'c',
     'Complex': 'FDG',
     'Datetime': 'Mm',
     'Float': 'fdg',
     'Integer': 'bhilqp',
    ...
    ```

  


| Home | Trees | Indices | Help | PyDSTool |
| --- | --- | --- | --- | --- |

|  |  |
| --- | --- |
| Generated by Epydoc 3.0.1 on Fri May 4 15:24:04 2012 | http://epydoc.sourceforge.net |
